# Risk of low serum levels of ionized magnesium in children with febrile seizure

**DOI:** 10.1186/s12887-018-1271-z

**Published:** 2018-09-07

**Authors:** Sung-Jin Baek, Jung Hye Byeon, So-Hee Eun, Baik-Lin Eun, Gun-Ha Kim

**Affiliations:** Department of Pediatrics, Korea University College of Medicine, Seoul, South Korea

**Keywords:** Seizure, Epilepsy, Magnesium, Child, Febrile

## Abstract

**Background:**

Suboptimal intake of magnesium become prevalent due to the modern diet of processed food low in magnesium. Magnesium may modulate seizure activity by antagonizing excitatory calcium influx through the N-methyl-D-aspartate receptor. Although hyponatremia has been reported to be common in febrile seizures, the most common form of seizure, little is known about the status of serum ionized magnesium. We therefore investigated the status of serum ionized magnesium (iMg^2+^) in children with febrile seizures and compared with controls.

**Methods:**

We included all patients from 1 to 6 years old who had presented with febrile seizure to the pediatric emergency department at the Korea University Guro Hospital from July 2016 to February 2017. The control group comprised patients admitted to the hospital with febrile respiratory tract infections, but with no history of febrile seizure. Clinical data, blood tests, and electroencephalogram (EEG) results were reviewed using the patients’ medical records.

**Results:**

A total of 133 patients with febrile seizure and 141 control patients were analyzed in the present study. As a result, hypomagnesemia (< 0.50 mmol/L) was more common in patients with febrile seizure than in controls (42.9% vs. 6.9%, *p* < 0.001) and it was an independent risk factor for febrile seizure (OR, odds ratio = 22.12, 95% CI = 9.23–53.02, *P* < 0.001). A receiver operating curve analysis revealed that serum iMg^2+^ levels < 0.51 mmol/L predicted the presence of febrile seizures with a sensitivity of 45.1% and a specificity of 92.6% (AUC, area under the curve = 0.731, 95% confidence interval = 0.671–0.791). When the patients with febrile seizure were divided in terms of a serum iMg^2+^ concentration of 0.51 mmol/L, there was no difference in clinical features.

**Conclusions:**

Hypomagnesemia was more common and serum iMg^2+^ level was lower in patients with febrile seizures than in controls. However, further evidence is needed for the causal relationship between low magnesium and febrile convulsions.

## Background

Magnesium is obtained from whole grains, nuts, and green leafy vegetables. However, global diet trends are moving towards high consumption of low-magnesium processed food [1, 2]. People who eat such diets are more likely to develop a magnesium deficiency, as are those who cook or boil all foods—especially vegetables, those who drink soft water, and those who eat food grown in magnesium-deficient soils, where synthetic fertilizers containing no magnesium are often used [1]. Furthermore, magnesium content can be reduced by 82–97% during the refining and processing of wheat to flour, rice to polished rice, or corn to starch [3]. In fact, large population surveys have shown that suboptimal magnesium intake is widespread [4–7] and that this may have a negative impact on human health.

Magnesium may modulate seizure activity by antagonizing excitatory calcium influx through the N-methyl-D-aspartate (NMDA) receptor [8–11]. Indeed, animal studies have shown that magnesium deficiency is related to increased seizure activity [12–14]. Magnesium sulfate has long been used to prevent eclampsia: a convulsive phase that follows pre-eclampsia in pregnant women, and recent studies have reported that magnesium can be used to treat several human epilepsies [15–17]. In febrile seizure, which afflicts 2–5% of the pediatric population and is the most common seizure disorder in children [18], only one third of patients have a positive family history of febrile seizure or epilepsy [19], and the majority of patients with febrile seizure have no evident risk factors. Although magnesium deficiency is fairly common, clinicians rarely test for or correctly measure it in patients with febrile seizure.

Clinicians can use the magnesium loading test to evaluate the body’s magnesium stores correctly [20, 21]. The preferred test is ionized magnesium instead of total magnesium, because less than 1% of the body’s magnesium is present extracellularly [22, 23].

In the present study, we hypothesized that serum magnesium levels could be lower in children with febrile seizures, the most common form of seizure, than in controls. Although hyponatremia has been reported to be common in febrile seizures, little is known about the status of serum ionized magnesium. We therefore investigated the status of serum ionized magnesium (iMg^2+^) in children with febrile seizures and compared with controls.

## Methods

### Patients and materials

The present study included all patients with febrile seizure between 1 and 6 years old who had presented to the pediatric emergency department at the Korea University Guro Hospital for 7 consecutive months from July 2016 to February 2017. Febrile seizure was described by caregivers as a convulsive event that was accompanied by fever (body temperature above 38 °C) in the hospital. We excluded patients with a history of afebrile seizures or an abnormal electroencephalography (EEG), as well as those whose iMg^2+^ levels had not been checked. Of the 152 patients who were screened, we excluded 10 who had a history of unprovoked seizure, one with abnormal EEG results, and eight whose serum iMg^2+^ level had not been checked. Overall, 133 patients were allocated into the febrile seizure group.

As a control group, we recruited consecutive patients who had been admitted with a febrile respiratory tract infection during the study period and whose serum iMg^2+^ levels had been checked. We gathered patients’ information and laboratory data using their medical records. Their levels of biochemical and hematologic analytes were included in the laboratory analysis. Serum iMg^2+^ levels were measured using the NOVA CRT8 analyzer (NOVA biomedical, USA).

### Statistical analysis

Data are presented as medians with inter-quartile ranges for non-normally distributed variables and as means ± standard deviations for normally distributed continuous variables. We used the student’s *t*-test for normally distributed variables, whereas the Mann–Whitney’s U-test was used for non-normally distributed variables to compare demographic and laboratory data between the febrile seizure and control groups. To assess the independent predictors of febrile seizure, univariate and multivariate logistic regression analyses were performed, with the results expressed as an odds ratio (OR) with a 95% confidence interval (CI). The diagnostic value of serum iMg^2+^ concentration was assessed using the area under the receiver operating characteristic (ROC) curve. All *p-*values less than 0.05 were considered statistically significant. Statistical analyses were performed using SPSS version 22.0 (IMB SPSS Inc., New York, United States).

## Results

### Characteristics of patients with simple febrile seizure

The characteristics of the febrile seizure group are shown in Table 1 (total: 133; 73 boys and 60 girls). Nine of these patients (6.8%) had multiple seizure episodes (five had two episodes and four had three episodes). Nineteen of the patients (14.3%) had a family history of febrile seizure, and four had a developmental delay (two motor and two language delays).

**Table 1:** Baseline characteristics of patients with febrile seizure (*n* = 133)

Men/Women	73 (54.9)/60 (45.1)
Age (year)	2.1 [1.7 – 3.1]
Family history of febrile seizure epilepsy	19 (14.3)/1 (0.8)
Past history of febrile convulsion	40 (30.1)
Developmental delay	4 (3.0)
Mean seizure duration (minute)	2.37 ± 2.20
Seizure longer than 15 min	0 (0)
Multiple seizure episodes	9 (6.8)
Seizure type (Focal/Generalized)	0 (0) / 133 (100)

Data are presented as medians [inter-quartile ranges] for non-normally distributed variables, as means ± standard deviations for all normally distributed continuous variables, and as n (%) in the case of countable variables. n, number of patients

### Comparison of laboratory values

One-hundred forty-one control patients who had been admitted with a febrile respiratory tract infection were compared with the 133 patients with febrile seizure (Table 2). Age and sex did not differ between the two groups, nor did the proportion of patients with abnormal laboratory values other than serum sodium and serum iMg^2+^. Hyponatremia was noted in 21.1% of the patients in the febrile seizure group and in 5.0% of those in the control group *(P* < 0.001). Hypomagnesemia was more common in the febrile seizure group than in the control group (42.9% vs. 6.9%, respectively; *P* < 0.001).

**Table 2:** Difference in laboratory data between patients with febrile seizures and control patients

Group	Febrile seizure (*n* = 133)	Control (*n* = 141)	*P*-value
Age (year)	2.1 [1.7 – 3.1]	1.9 [1.5 – 3.0]	0.128
Men/Women	73 (54.9)/60 (45.1)	77 (54.6)/64 (45.4)	1.000
Hypocalcemia^a^	13 (11.6)	14 (10.6)	0.763
Hypomagnesemia	57 (42.9)	7 (6.9)	< 0.001*
Serum iMg^2+^ (mmol/L)	0.5 ± 0.1	0.6 ± 0.0	
Hyponatremia	28 (21.1)	7 (5.0)	< 0.001*
Serum Na (mmol/L)	136.0 [135.0;137.0]	138.0 [137.0; 139.0]	
Hypokalemia	3 (2.3)	5 (3.5)	0.783

Data are presented as medians [inter-quartile ranges] for non-normally distributed variables, as means ± standard deviations for all normally distributed continuous variables, and as n (%) in the case of countable variables. Normal pediatric ranges as follows: Ca (9.2–10.6 mg/dL), iMg^2+^ (0.50–0.70 mmol/L), Na (135–145 mmol/L), and K (3.6–5.2 mmol/L). ^a^Total calcium levels were not tested in several patients (21 in the febrile seizure group and nine in the control group). n, number of patients. ^*^Significant

### Predicting independent risk factors for developing febrile seizure

We performed univariate and multivariate logistic regression analysis to determine independent demographic and laboratory risk factors for developing febrile seizure (Table 3). The analysis revealed that hypomagnesemia and hyponatremia were independent risk factors (OR = 22.12, 95% CI = 9.23–53.02, *P* < 0.001 and OR = 4.81; 95% CI = 1.67–13.85, *p* = 0.0036, respectively).

**Table 3:** Univariate and multivariate analysis of significant risk factors associated with febrile seizure (*n* = 274)

	Univariate Analysis			Multivariate Analysis		
	Crude OR	95% CI	*P*-value	Adjusted OR	95% CI	*P*-value
Age	1.09	0.89–1.34	0.4189			
Sex	0.99	0.61–1.59	0.9632			
Hypocalcemia	1.23	0.55–2.75	0.6120			
Hypomagnesemia	14.36	6.24–33.05	< 0.001	22.12	9.23–53.02	< 0.001
Hyponatremia	5.10	2.15–12.14	0.0002	4.81	1.67–13.85	0.0036
Hypokalemia	0.63	0.15–2.68	0.5294			

Normal pediatric ranges as follows: Ca (9.2–10.6 mg/dL), iMg^2+^ (0.50–0.70 mmol/L), Na (135–145 mmol/L), and K (3.6–5.2 mmol/L). n, number of patients; OR, odds ratio; CI, confidence interval; iMg^2+^, Ionized magnesium

### Hypomagnesemia

**Fig. 1:**
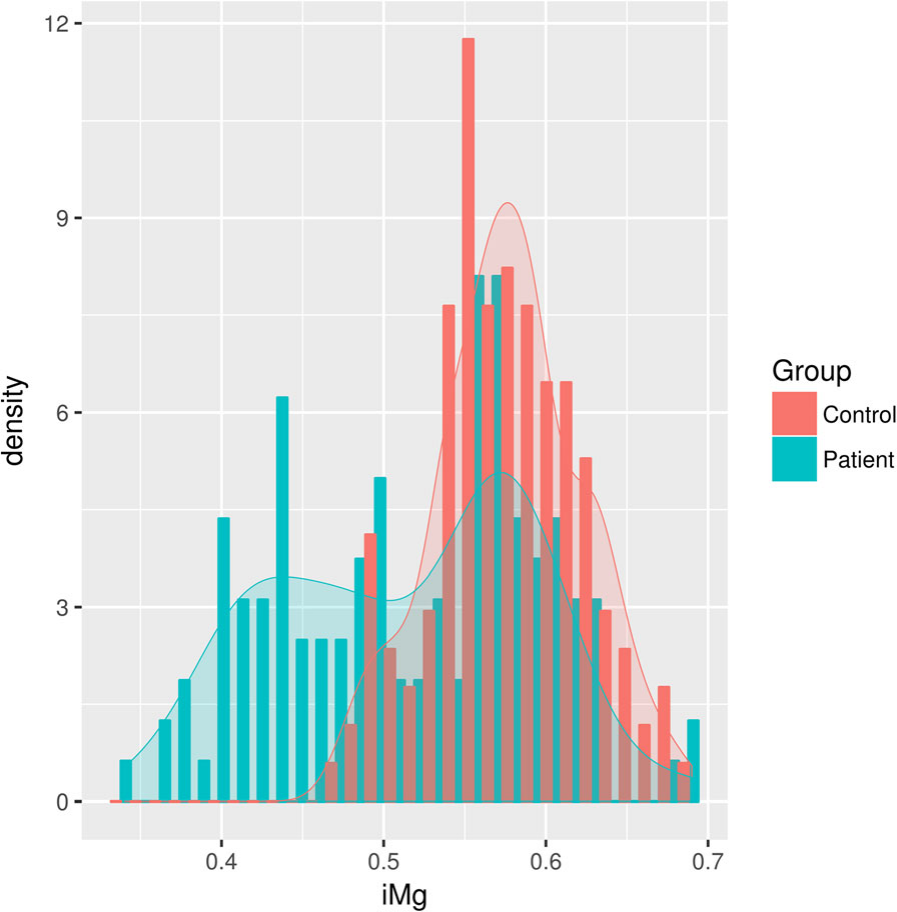
Density plot graphic of serum iMg^2+^ levels in the patients with febrile seizures and in controls. Serum iMg^2+^ levels in patients with febrile seizures are lower than in control patients (mean ± SD: 0.5 ± 0.1 vs. 0.6 ± 0.0 mmol/L, respectively; *P* < 0.001)

**Fig. 2:**
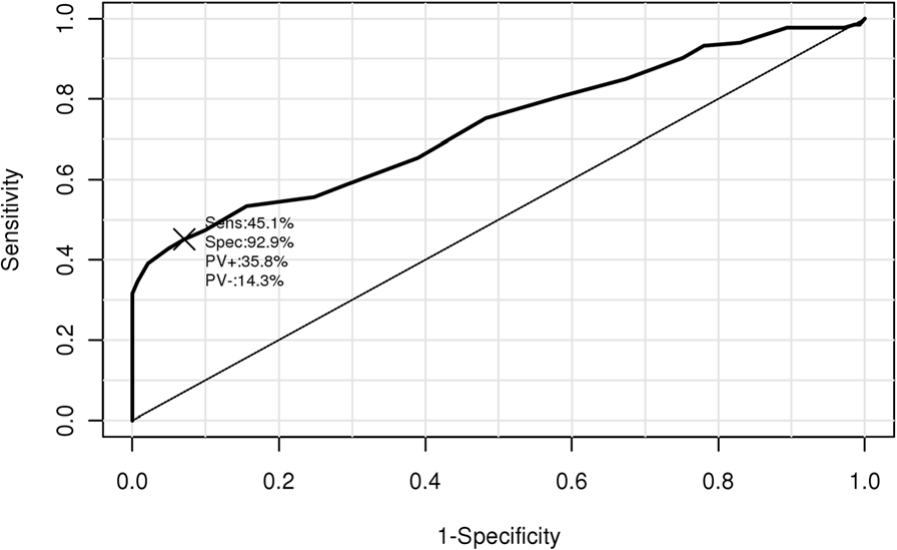
Receiver operating curve analysis of serum iMg^2+^ levels in the febrile seizure and control groups (cut-off level: 0.51 mmol/L, sensitivity: 45.1%, specificity: 92.9%; area under curve: 0.731, 95% confidence interval: 0.671–0.791). Sens, sensitivity; Spec, specificity; PV+, positive predictive value; PV-, negative predictive value

As shown in Fig. 1, serum iMg^2+^ levels were lower in patients with febrile seizure than in control patients (mean ± SD = 1.0 ± 0.2 vs. 1.2 ± 0.1 mEq/L, respectively; *P* < 0.001). The area under the ROC curve for serum iMg^2+^ levels was 0.731 (95% CI = 0.671–0.791), indicating that serum iMg^2+^ levels of < 0.51 mmol/L in children predicted the presence of febrile seizures with a sensitivity of 45.1% and a specificity of 92.9% (Fig. 2).

As noted in Table 4, we next divided the patients with febrile seizure into two subgroups on the basis of serum iMg^2+^ levels (< 0.51 mmol/L and ≥ 0.51 mmol/L). We found no difference between the groups in terms of seizure duration or number of seizure episodes.

**Table 4:** Comparison of clinical variables according to ionized magnesium level among patients with febrile seizure

Variables	Serum levels of ionized Mg^2+^		*P*-value
	< 0.51 mmol/L (*n* = 60)	≥ 0.51 mmol/L (*n* = 73)	
Men/Women	33 (55.0)/27 (45.0)	40 (54.8)/33 (45.2)	1.000
Age (year)	2.2 [1.7; 3.4]	2.1 [1.7; 3.0]	0.741
Family history of epilepsy	0	1 (1.4)	1.000
Family history of FS	10 (16.7)	9 (12.3)	0.644
Past history of FS	18 (30.0)	22 (30.1)	1.000
Developmental delay	0	4 (5.5)	0.183
Multiple seizure episodes	5 (8.3)	4 (5.5)	0.760
Seizure duration (min)	2.0 [1.0; 3.0]	1.0 [1.0; 3.0]	0.366
Seizure longer than 5 min	2 (3.3)	5 (6.8)	0.608

Data are presented as medians [inter-quartile ranges] for non-normally distributed variables, as means ± standard deviations for all normally distributed continuous variables, and as n (%) in the case of countable variables. n, number of patients; FS, febrile seizure

## Discussion

In clinical practice, hypomagnesemia is underdiagnosed or incorrectly measured in patients with febrile seizure. People with modern diets are more likely to have low magnesium stores in their body [1–7], and magnesium can modulate seizures [8–14]. For these reasons, it could be beneficial to accurately measure iMg^2+^ levels, not total magnesium levels, in patients with febrile seizures.

In the present study, we found that hypomagnesemia and mild hyponatremia were more common in patients with febrile seizure than in those with febrile respiratory tract infection (Fig. 1). Multivariate logistic regression analysis revealed that both hypomagnesemia (< 0.50 mmol/L) and hyponatremia (< 135 mmol/L) were independent risk factors for developing febrile seizure. Since few studies have addressed iMg^2+^ levels in children, we determined an optimal cut-off value for the occurrence of febrile seizures. An ROC analysis revealed that serum iMg^2+^ levels < 0.51 mmol/L in children predicted the presence of febrile seizures with a sensitivity of 45.1% and a specificity of 92.9% (area under the ROC curve = 0.731, 95% CI = 0.671–0.791; Fig. 2).

### Literature about hyponatremia and hypomagnesemia in febrile seizure

Many studies have noted that hyponatremia is common in cases of febrile seizure [20, 21, 24–28]. Besides, several studies have claimed that hyponatremia can predict further seizures [20, 24, 25], while others have contradicted this finding [21, 26–28]. One investigation reported that patients with hyponatremic febrile seizure had increased arginine vasopressin levels on the first day of admission, and that they had decreased sodium and osmolality levels on the second day. These findings suggest that fever and other non-osmotic stimuli lead to excess arginine vasopressin, causing transient mild hyponatremia [29]. Mild hyponatremia in our study was usually measured immediately after febrile seizure in the emergency room, thus, which is thought to be due to febrile illness or mild dehydration rather than excessive arginine vasopressin secretion.

For hypomagnesemia, only one previous investigation related to febrile seizure reported that hypomagnesemia was noted in 86% of patients with simple febrile seizure [30]. However, the study has limited value because of that they measured the total magnesium rather than the iMg^2+^ concentration in the blood and did not involve a control group.

### Anti-seizure effect of magnesium

It may be that magnesium acts as an anticonvulsant because it modulates seizure activity by antagonizing excitatory calcium influx through the NMDA receptors [31–34].

In particular, magnesium sulfate increases the seizure threshold without affecting the permeability of the blood–brain barrier in a rat model of severe pre-eclampsia [35]. A single oral dose of magnesium can inhibit NMDA-induced convulsions in mice in a dose-dependent manner [34], and Continuous intravenous magnesium infusion reduced seizures in one of two patients with fever-related epilepsy syndrome [16]. Furthermore, in patients with infantile spasms, combined treatment using adrenocorticotropic hormone and intravenous magnesium sulfate yielded a better response than treatment using adrenocorticotropic hormone alone (79% vs. 53%) [17]. Finally, one study found that oral magnesium was an effective adjunct treatment for medically intractable epilepsies [36], reporting that 36% (8/22) of the patients saw a ≥ 75% reduction in the number of seizure days per month after a follow-up of 6–12 months.

### Measurement of physiologically active magnesium concentration

Most magnesium is stored within bones (50%) and soft tissues (47%), while less than 1% of total body magnesium is present in blood, with approximately 0.3% in serum [22, 23]. Thus, clinicians should use the magnesium loading test to evaluate the body’s magnesium stores correctly [20, 21] or measure the free, ionized form of magnesium, which is physiologically active. One study found that, in 25% of patients, biologically active levels of iMg^2+^ were not reflected in an analysis of total magnesium, and that iMg^2+^ was only weakly correlated with total magnesium [37].

In 1992, Altura et al. designed a novel ion selective electrode that utilizes a neutral carrier-based membrane to assess iMg^2+^ in whole blood, plasma, and serum [38], so iMg^2+^ levels can now be easily measured. Nevertheless, most clinical laboratories still measure total magnesium levels using colorimetry or atomic absorption spectrophotometry.

### Reference interval of ionized magnesium level

The iMg^2+^ level varies according to the analytical method, instrument type, matrix, and reagent composition used [39]. Several reports have estimated the reference interval of iMg^2+^ in adults using the NOVA CRT8. The mean iMg^2+^ level in healthy Canadian adults was 0.52 (range: 0.44–0.59) mmol/L in whole blood [40], while it was 0.57 ± 0.03 (range: 0.51–0.63) mmol/L in Japanese adults. However, only a few reports have measured iMg^2+^ levels in children using the NOVA CRT8. One by Hoshiono et al. involved 160 healthy Japanese children and found that the mean iMg^2+^ level was 0.53 ± 0.03 (range: 0.45–0.63) mmol/L [41].

Our study had several limitations. By selecting patients with respiratory infections with fever as a control group, it is advantageous to eliminate the effects of fever on magnesium concentrations in the blood. However, it is also meaningful to compare three groups, including healthy controls. Furthermore, when analyzing the risk factors for febrile seizure, we assumed that the magnesium concentration after the seizure was the same as that before. However, this may not have been the case.

## Conclusions

Hypomagnesemia was more common and serum iMg^2+^ levels were lower in patients with febrile seizures than in controls. However, further evidence is needed for the causal relationship between low magnesium and febrile convulsions.

## Abbreviations

EEG: Electroencephalography
iMg^2+^: Ionized magnesium
NMDA: N-methyl-D-aspartate
